# Draft Genome Sequence of *Frankia* sp. Strain BMG5.23, a Salt-Tolerant Nitrogen-Fixing Actinobacterium Isolated from the Root Nodules of *Casuarina glauca* Grown in Tunisia

**DOI:** 10.1128/genomeA.00520-14

**Published:** 2014-05-29

**Authors:** Faten Ghodhbane-Gtari, Sheldon G. Hurst, Rediet Oshone, Krystalynne Morris, Feseha Abebe-Akele, W. Kelley Thomas, Amir Ktari, Karima Salem, Maher Gtari, Louis S. Tisa

**Affiliations:** a Laboratoire Microorganismes et Biomolécules Actives, Université Tunis El Manar (FST) & Université Carthage (INSAT), Campus Universitaire, Tunis, Tunisia; b University of New Hampshire, Durham, New Hampshire, USA

## Abstract

Nitrogen-fixing actinobacteria of the genus *Frankia* are symbionts of woody dicotyledonous plants termed actinorhizal plants. We report here a 5.27-Mbp draft genome sequence for *Frankia* sp. strain BMG5.23, a salt-tolerant nitrogen-fixing actinobacterium isolated from root nodules of *Casuarina glauca* collected in Tunisia*.*

## GENOME ANNOUNCEMENT

Soil-dwelling nitrogen-fixing actinobacteria of the genus *Frankia* are best known for their symbiotic lifestyle with over 200 species of dicotyledonous plants termed actinorhizal plants (1–3). As ecologically important pioneer community plants, actinorhizal plants are distributed worldwide in a broad range of ecological and environmental conditions. Besides their ecological role, these plants have economic significance in land reclamation, reforestation, soil stabilization, dune stabilizers, and fuel wood. The symbiosis allows actinorhizal plants to colonize harsh environmental terrains under diverse ecological conditions. Molecular phylogenetic approaches have identified four distinct clusters among the *Frankia* strains (4–7). Genomes for representatives from four major lineages have been sequenced (8–14) and have provided vital baseline information for genomic approaches toward understanding these novel bacteria.

Under tropic and subtropic conditions, actinorhizal plants are essentially represented by fast growing and highly tolerant trees from the family *Casuarinaceae*. Since the end of the 19th century, these plants have been exported from their natural habitat in Australia and Western Pacific Islands to worldwide locations for agroforestery systems, essentially serving as windbreaks, dune stabilizers, fuel wood, and soil regeneration (15). *Frankia* sp. strain BMG5.23 was isolated from root nodules of *Casuarina glauca* growing in Tunisia and effectively re-infects its original host plant, *Casuarina* spp. (7). Based on analysis of ITS 16S-23S rRNA (5) and *gyrB*, *glnII*, and *nifH* (6) gene sequences, *Frankia* sp. strain BMG5.23 groups with the “*Casuarina* infective strains” of cluster 1. This strain showed an increased level of NaCl tolerance (R. Oshone and L. S. Tisa, unpublished data). *Frankia* sp. strain BMG5.23 has the potential to be used as a large scale inoculum for *Casuarina* trees involved in land reclamation of Tunisian saline soils. *Frankia* sp. strain BMG5.23 was sequenced to increase our understanding of the salt-tolerance mechanisms and to provide information about its potential ecological roles and interaction with actinorhizal plants.

The draft genome of *Frankia* sp. strain BMG5.23 was generated at the Hubbard Genome Center (University of New Hampshire, Durham, NH) using Illumina technology (16) techniques. A standard Illumina shotgun library was constructed and sequenced using the Illumina HiSeq2000 platform, which generated 18,742,322 reads (260 bp insert size) totaling 1,695.5 Mbp. The Illumina sequence data were assembled using CLC Genomics Workbench (6.5.1) and AllPaths-LG (version r41043) (17). The final draft assembly contained 167 contigs with an *N*_50_ of 64.9 kb. The total size of the genome is 5.3 Mbp, and the final assembly is based on 1,533.9 Mb of Illumina draft data, provided an average 291.2× coverage of the genome.

The high quality draft genome of *Frankia* sp. strain BMG5.23 was resolved to 167 contigs consisting of 5,267,418 bp with a G+C content of 69.8%. The assembled *Frankia* sp. strain BMG5.23 genome was annotated via the Integrated Microbial Genomes (IMG) platform developed by the Joint Genome Institute, Walnut Creek, CA (18), and resulted in 4,747 candidate protein-encoding genes, 47 tRNA genes and 3 rRNA regions.

### Nucleotide sequence accession numbers.

This whole-genome shotgun project has been deposited at DDBJ/EMBL/GenBank under the accession no. JDWE00000000. The version described in this paper is version JWDE01000000.
